# Roles of mesenchymal stem cells and exosomes in interstitial cystitis/bladder pain syndrome

**DOI:** 10.1111/jcmm.17132

**Published:** 2021-12-24

**Authors:** Chao Wen, Liping Xie, Chenxia Hu

**Affiliations:** ^1^ Department of Urology School of Medicine The First Affiliated Hospital Zhejiang University Hangzhou China; ^2^ State Key Laboratory for the Diagnosis and Treatment of Infectious Diseases Collaborative Innovation Center for the Diagnosis and Treatment of Infectious Diseases School of Medicine The First Affiliated Hospital Zhejiang University Hangzhou China; ^3^ National Clinical Research Center for Infectious Diseases School of Medicine The First Affiliated Hospital Zhejiang University Hangzhou China

**Keywords:** bladder pain syndrome, dysfunction, exosome, interstitial cystitis, mesenchymal stem cell

## Abstract

Interstitial cystitis/bladder pain syndrome (IC/BPS) is characterized by several symptoms of higher sensitivity of the lower urinary tract, such as bladder pain/discomfort, urgency, urinary frequency, pelvic pain and nocturia. Although the pathophysiology of IC/BPS is not fully understood, the hypothesis suggests that mast cell activation, glycosaminoglycan (GAG) layer defects, urothelium permeability disruption, inflammation, autoimmune disorder and infection are potential mechanisms. Mesenchymal stem cells (MSCs) have been proven to protect against tissue injury in IC/BPS by migrating into bladders, differentiating into key bladder cells, inhibiting mast cell accumulation and cellular apoptosis, inhibiting inflammation and oxidative stress, alleviating collagen fibre accumulation and enhancing tissue regeneration in bladder tissues. In addition, MSCs can protect against tissue injury in IC/BPS by secreting various soluble factors, including exosomes and other soluble factors, with antiapoptotic, anti‐inflammatory, angiogenic and immunomodulatory properties in a cell‐to‐cell independent manner. In this review, we comprehensively summarized the current potential pathophysiological mechanisms and standard treatments of IC/BPS, and we discussed the potential mechanisms and therapeutic effects of MSCs and MSC‐derived exosomes in alleviating tissue injury in IC/BPS models.

## 1 INTRODUCTION

Interstitial cystitis/bladder pain syndrome (IC/BPS) is an umbrella term of chronic debilitating conditions characterized by symptoms of lower urinary tract hypersensitivity that lead to poor quality of life in IC/BPS patients. Although it is not easy to evaluate the true prevalence of IC/BPS according to the heterogenic definitions and nomenclature, approximately 3%–7% prevalence is reported according to current studies.^1^ These patients always complain of chronic clinical symptoms such as bladder pain/discomfort, pelvic pain, urinary frequency, urgency and nocturia, but there is no bacterial infection in the urinary system after laboratory examinations.^2^ Cameron et al. demonstrated that some women with IC/BPS also had several complaints of bladder outlet obstruction (BOO) with sensation of incomplete emptying, slow stream, straining to void and dribbling. They postulated that painful voiding was at the root of the problem with pain causing reflexive poor pelvic floor relaxation.^3^ In addition, specific individuals have accompanying symptoms, including chronic stress, sleep dysfunction, anxiety and sexual dysfunction.^4^ The clinical diagnosis of IC/BPS is based on these typical symptoms after exclusion of other alternative diseases with a similar presentation, including urinary tract infection, neoplasia and bladder calculi. In more recent years, IC/BPS has been reported to be closely related to other systemic pain syndromes, including chronic fatigue syndrome, fibromyalgia and irritable bowel syndrome.^5^

Stem cell‐based therapy has emerged as a valuable treatment to protect against tissue injury in different diseases. Stem cells can be classified into various cell types, such as mesenchymal stem cells (MSCs), embryonic stem cells (ESCs), induced pluripotent stem cells (iPSCs) and hematopoietic stem cells.^6^ In comparison with ESCs and iPSCs, MSCs have less tumorigenicity in vivo, which enables them to be safer in clinical treatments. MSCs can be isolated from different adult tissues such as umbilical cord blood, bone marrow, adipose tissue, placenta, amniotic fluid, urine and other tissues.^7^, ^8^, ^9^, ^10^, ^11^ They express low levels of major histocompatibility complex (MHC) class I molecules and MHC class II molecules, which enable them to have low immunogenicity.^12^ In vitro, MSCs can differentiate into a range of cell types, including adipocytes, osteocytes, hepatocytes, chondrocytes, muscle, vascular smooth muscle cells and other connective tissues.^13^, ^14^, ^15^ In response to injury signals, MSCs migrate into damaged target tissues^16^, ^17^ and differentiate into local progenitor cells to replace ageing cells or apoptotic cells in vivo.^18^, ^19^ In addition, the cytokines and growth factors secreted by MSCs yield beneficial outcomes in injured tissues or organs by stimulating angiogenesis and proliferation of endogenous progenitor cells but alleviating inflammatory reactions and tissue fibrosis. These paracrine effects are considered the primary therapeutic mode of action for these cells in some respects.^20^ All of these mechanisms enable MSCs and their exosomes to be potential therapeutic agents for IC/BPS according to current studies.

In this review, we comprehensively summarized the current potential pathophysiological mechanisms and standard treatments of IC/BPS, and we discussed the potential mechanisms and therapeutic effects of MSCs and MSC‐derived exosomes in alleviating tissue injury in IC/BPS models.

## 2 THE PATHOPHYSIOLOGICAL MECHANISMS OF IC/BPS

**FIGURE 1:**
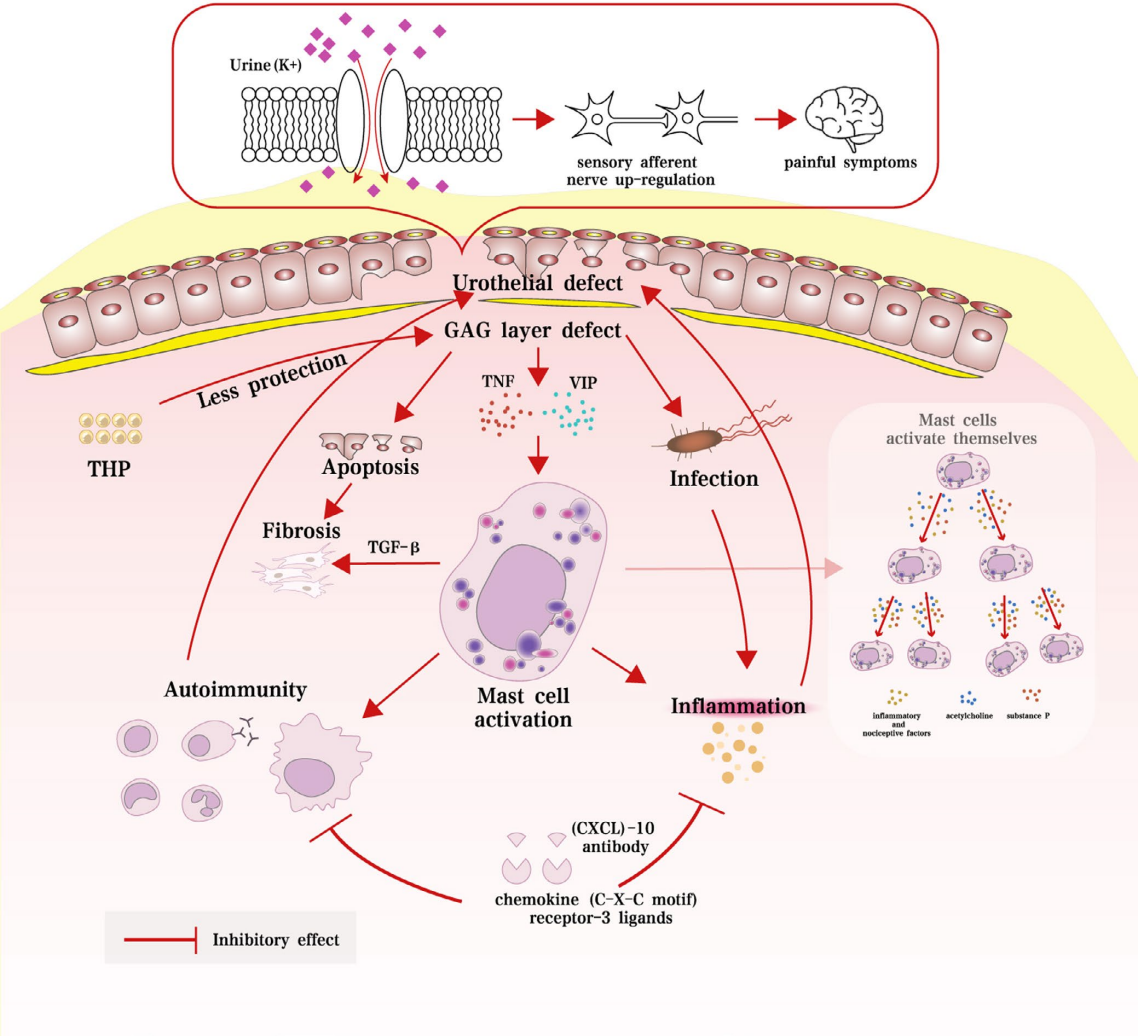
Potential pathophysiological mechanisms of IC/BPS according to current studies

Although the pathophysiological mechanisms are not identifiable and not fully understood, a hypothesis suggests that mast cell activation, glycosaminoglycan (GAG) layer defects, urothelium permeability disruption, inflammation, autoimmune disorder and infection are potential mechanisms (Figure 1). The urothelium in bladder tissue is covered by mucopolysaccharides and consists of a basal cell layer, an intermediate layer and a superficial layer. The urothelium provides a barrier to defend the underlying tissues against urine and create a selected and specific movement of molecules towards the bladder wall.^21^ The umbrella cells were denudated to develop defects in integrity and microplicae and severe pleomorphism in patients with IC/BPS.^22^

Injury factors or allergic reactions induce urothelium dysfunction and result in secreting different inflammatory cytokines (vasoactive intestinal peptide [VIP] and tumour necrosis factor [TNF]), which subsequently activate mast cells.^23^ Moreover, the specifically activated mast cells secrete a group of inflammatory and nociceptive factors and neurotransmitters (acetylcholine and substance P), which further promote the stimulation of other mast cells.^24^ The bladder surface mucus secretes a hydrophilic GAG layer that traps water at the outer layer to form a key protective barrier at the important space between the urine and the bladder.^25^ GAG molecules consist of a family of polysaccharide molecules and play critical roles in the regulation of enzymes, growth factors, protease inhibitors and cytokines. GAGs, fibronectin, elastin, collagen and laminin are known to form the basic framework of the extracellular matrix.^21^ However, disruption of the GAG layer by inflammation, infection, damage, lesions and other factors results in upregulation of urothelial permeability and imbalance in urine storage.^26^ Tamm‐Horsfall protein (THP) is the most abundant protein that is protective in human urine and terminates in sialic acid,^27^, ^28^ while the THP of IC/BPS patients is less protective against urinary cations that can injure the mucus of the urothelium.^29^ Potassium sensitivity is also a key mechanism in the progression of IC/BPS, since protamine sulfate administration can induce potassium absorption and injury to the bladder surface mucus.^30^ The upregulation of influx of potassium ions and urothelial permeability leads to the activation of sensory afferent nerves in a vicious cycle, which contributes to the painful symptoms of IC/BPS.^31^, ^32^ Neurogenic inflammation contributes to bladder afferent hypersensitivity and stromal fibrotic alterations via nerve‐mast cell interactions.^33^ Moreover, IC/BPS patients were found to have increased angiogenesis, glomerulation formation and urothelial deficiency.^22^ Intriguingly, a relationship between IC/BPS and other autoimmune diseases has been reported. A large number of T cells, monocytes, mast cells, B cells and plasma cells infiltrate the urinary bladders of IC/BPS patients,^34^, ^35^ and the ratio of inflammatory cells and T helper cells to suppressor cells is upregulated in the peripheral blood of these patients.^36^ An anti‐C‐X‐C motif chemokine ligand (CXCL)‐10 antibody effectively alleviated the severity of chronic IC/BPS by locking chemokine (C‐X‐C motif) receptor‐3 ligands and reducing the release of proinflammatory cytokines and infiltrated immune cells.^37^ Upon exposure to bacterial infection, the symptoms were exacerbated, and the expression of E‐cadherin was downregulated in the urothelium.^38^ Removal of mucus with an acidic solution or detergents results in a significant increase in bacterial adherence or bacterial infection.^39^ Although various studies considered infection a potential mechanism of IC/BPS, excluding infection of the lower urinary tract was highlighted as a diagnostic criterion of IC/PBS.^40^ This opinion raised the possibility that current infection should be excluded in the diagnosis of IC/BPS, while previous infection‐induced urothelium injury and inflammatory response may act as a potential mechanism in the progression of IC/BPS. According to current studies, urothelium injury induced by different factors is the key point in the pathophysiological process of IC/BPS.

## 3 STANDARD TREATMENTS FOR IC/BPS

Targeted treatments have mixed outcomes, since the mechanisms are complex and the complicated symptoms are not easy to control. Specific treatments that target the related mechanisms are recommended. IC/BPS patients have a relatively low quality of life because of unmanageable symptoms, and the main target is to improve the quality of life of IC/BPS patients.^41^ Behavioural, physical, pharmacological, endoscopic and surgical therapies must be simultaneously considered to synergistically improve the quality of life of patients.

The first step in the management of patients with IC/BPS includes conservative and behavioural guidance, including timed voiding and bladder training, which serve as the foundation for long‐term treatment because they are risk‐free and relatively inexpensive for all patients.^42^ Physiotherapy includes soft tissue massage of the pelvic floor, and myofascial trigger point release is also a good option for patients with pelvic floor dysfunction and trigger point or myofascial tenderness.^43^

Pharmacotherapy includes a wide range of drugs and is currently applied in the clinic via the oral or intravesical route, while a few of them are recommended because of heterogeneity. Although amitriptyline is not licensed for application in IC/BPS, this tricyclic antidepressant is used to effectively alleviate neuropathic pain when patients can tolerate a dose of at least 50 mg.^44^ Pentosan polysulfate is a licensed drug for the treatment of IC/BPS. Van et al.^45^ demonstrated that pentosan polysulfate effectively alleviated discomfort symptoms such as pain, urgency and frequency compared with the placebo, and pentosan polysulfate could replace the GAG layer and inhibit mast cell degranulation.^46^ Antihistamines benefit from preventing histamine release from mast cells and are potential therapeutic agents in IC/BPS patients, but evidence of efficacy is conflicting and based on low‐quality studies.^47^ Cyclosporin A targets T‐cell activation and cytokine release and has emerged as a treatment option for IC/BPS patients. The application should be cautious because it may exert severe side effects. It is preferable for patients with refractory BPS who are not sensitive to other oral or intravesical agents.^48^ Intravesical dimethylsulfoxide (DMSO) demonstrated high efficacy in symptom improvements and urodynamic and voiding diary changes with minor side effects.^49^ In addition, DMSO remains the only approved drug by the Food and Drug Administration (FDA) to treat IC/BPS, and it is hypothesized to inhibit inflammatory mast cells and promote the relaxation of bladder muscles.^50^ Intravesical pentosan polysulfate was reported to more significantly increase the efficacy of pentosan polysulfate in alleviating IC/BPS symptoms than oral pentosan polysulfate.^51^ Intravesical lidocaine is another optional treatment with 30%–50% symptom improvements in IC/BPS.^52^ Intravesical GAG replenishment therapies focus on restoring the GAG layer lining the urothelium. Hyaluronic acid and chondroitin sulfate are effective in palliation of symptoms, while the efficacy is generally limited.^53^, ^54^ Intravesical botulinum toxin type A could block the release of neurotransmitters from bladder afferent nerve fibres, subsequently alleviated the sensitivity and enhanced the detrusor contractility in IC/BPS.^55^ Pharmacotherapy targets each mechanism of IC/BPS, while the efficacy should be improved, and the side effects should be decreased in future studies.

Bladder distention has served as both treatment and diagnostic tool for IC/BPS patients for many years, while the technique has an unverified outcome because of the heterogeneity in patient selection and hydrodistension techniques.^56^ Meanwhile, the implanted electrode and conducting lead in sacral neuromodulation stimulate the afferent sacral nerves when they exit the sacral foramina, which obviously alleviates symptoms of pelvic pain, frequency, nocturia and urgency in IC/BPS patients.^57^ However, some patients with implantation of the device found that the symptoms were aggravated, which indicates that revision or explanation of the device is necessary.

Radical surgery is the last resort option for severe and disabling IC/BPS patients who are not sensitive to other treatments. Most recently, in 2021, a systematic review concluded that 77% of 448 IC/BPS patients had symptomatic improvement after they underwent radical surgery, and total cystectomy with orthotopic neobladder formation had the highest clinical response compared to subtotal cystectomy with cystoplasty and urinary diversion alone.^58^

Although there are various treatment tools for IC/BPS patients, there are still no definite treatments in terms of recurrence, side effects or lack of response. IC/BPS is a chronic condition that may be controlled but not cured. It is necessary to investigate new and safer agents to inhibit pathophysiological processes.

## 4 MSCs AND IC/BPS

Although there have been no definitive treatments to cure this heterogeneous disease until now, MSCs have emerged as a new therapeutic agent in IC/BPS. Bladder dysfunction is an excellent candidate for stem cell therapy due to its chronic nature and high prevalence.^59^ Compared to radical surgery, MSC transplantation is easier to manipulate. In comparison with pharmacological agents, they are pleiotropic and have no severe side effects. MSCs can be isolated from autologous tissue without rejection reaction. Moreover, cell‐free therapy based on MSC‐derived exosomes is an emerging treatment that benefits from abundant anti‐inflammatory and anti‐immune cytokines.

### 4.1 MSCs effectively alleviated tissue injury of IC/BPS via different mechanisms

**FIGURE 2:**
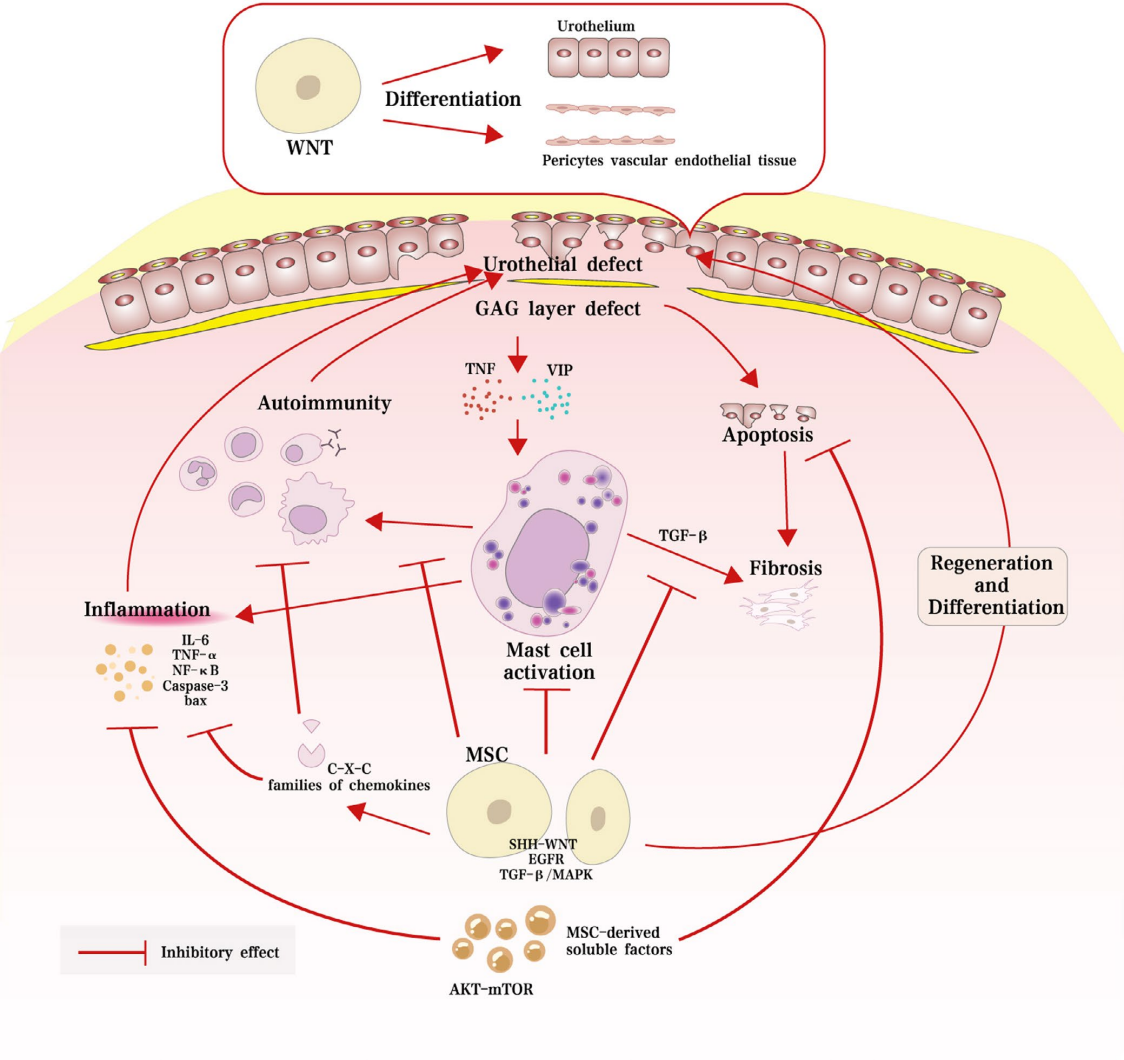
Underlying mechanism of MSCs and their derivatives in protecting against tissue injury in IC/BPS

Mesenchymal stem cells have been proven to protect against tissue injury in IC/BPS via different mechanisms (Table 1). MSCs are able to migrate into bladders, differentiate into key bladder cells, inhibit mast cell accumulation and cellular apoptosis, decrease the release of inflammatory cytokines and oxidants, alleviate the deposition of collagen fibres and enhance tissue regeneration in bladders (Figure 2).

**TABLE 1:** Potential mechanisms of MSCs from diverse tissues in alleviation of tissue injury in IC/BPS

Species	Tissue origin	Dose (MSC)	Control	Route	Model	Injury factor	Effect	Mechanism	Ref.
Human	Urine; adipose; bone marrow; amniotic fluid	1 × 10^5^	PBS	Bladder mucosa	Rat	Uroplakin II	Regenerate damaged bladder tissue; promote functional recovery; inhibit inflammatory cell accumulation	Inhibit the expression of MPO, IL−1β, IL−6, IL−17α, TNF‐α, TLR4, TLR5 and TLR11	^11^
Human	Embryonic stem cell	1 × 10^6^	PBS	Bladder serosa	Rat	PS/LPS	Decrease bladder voiding function and the pathological characteristics of the bladder	Engraft and differentiate into multiple cell types; integrate into a perivascular‐like structure	^61^
Human	Embryonic stem cell	1 × 10^6^	PBS	Outer layer of the anterior wall and dome of the bladder	Rat	HCl	Ameliorate bladder symptoms; ameliorate bladder voiding function and histological injuries	Decrease urothelium denudation, mast cell infiltration, tissue fibrosis, apoptosis, and visceral hypersensitivity; integrate into vascular‐like structures	^62^
Human	Umbilical cord blood	1 × 10^6^	PBS	Submucosal layer of the bladder	Rat	HCl	Attenuate the irregular and decreased voiding interval	Engraft to the stromal and epithelial tissues; activate Wnt signalling cascade; prevent denudation of the epithelium and increased inflammatory responses, mast cell infiltration, neurofilament production, and angiogenesis	^63^
Human	Dental pulp	2 × 10^6^	PBS	Bladder	Rat	Hydrochloride	Alleviate the submucosal oedema and haemorrhage; prolong intercontraction interval; reduce the frequency of nociceptive behaviours	Decrease the concentrations of myeloperoxidase and the levels of inflammatory cytokines; increase the levels of FGF−2, VEGF, and the C‐C and C‐X‐C families of chemokines	^64^
Human	Umbilical cord	1 × 10^6^	PBS	Tail vein	Rat	CYP	Ameliorate the bladder voiding function	Repair impaired bladder; reduce mast cell infiltration; inhibit apoptosis of urothelium; decrease the levels of IL−1β, IL−6 and TNF‐α in bladder; promote the proliferation of impaired uroepithelial cells; inhibit cellular apoptosis	^65^
Human	Urine	1.2 × 10^6^	Saline	Bladder	Rat	PS/LPS	Improve the bladder micturition function	Upregulate the expression levels of antioxidants and antiapoptotic proteins; decrease the levels of autophagy‐related protein LC3A and inflammatory factors	^67^
Human	Umbilical cord blood	1 × 10^6^	PBS	Submucosal layer of bladder	Rat	Ketamine	Repair the damaged voiding function; decrease voiding frequencies; increase intercontraction intervals; reduce contraction pressure	Protect against an abnormal alteration in mast cell infiltration, apoptosis, and fibrotic damage	^68^
Rat	Adipose	1 × 10^6^	PBS	Anterior and posterior bladder walls	Rat	HCl	Decrease nociceptive behaviour; increase bladder capacity and voiding threshold pressures	Decrease the expression of TNF‐α, TGF‐β, and collagen fibres	^69^
Rat	Bone marrow	1 × 10^5^	Bladder transplantation + PBS injection	Tunica muscularis vesicae urinariae	Rat	Protamine sulfate	Increase discharge volume, maximal micturition volume, contraction interval, and bladder capacity; decrease residual urine volume, bladder pressure, and bladder peak pressure	Decrease expression of TGF‐β/MAPK signalling pathway‐related proteins; decrease levels of inflammatory cytokines	^70^
Rat	Bone marrow	1 × 10^6^	Unobstructed control	Intravenously injection	Rat	Urethral ligation	Reduce the urine storage pressure, hypertrophy, and fibrosis	Reduce the level of TNF‐α; increase the levels of IL−10 and VEGF	^72^
Rat	Bone marrow	4 × 10^6^	Sham operation	Unilateral side of the iliac artery	Rat	Bilateral iliac artery ligation	Increase intravesical pressure and detrusor contraction power	Increase smooth muscle content and nerve fibre content; regenerate in the bladder tissue	^73^

After MSCs are injected into the submucosal layer of the bladder, they are colocalized with both epithelial and stromal cells and mostly distributed in the lamina propria but less in the muscular layer. Then, they directly differentiate into urothelial cells and nearby stromal tissue. In addition to the differentiation capacity, MSC transplantation reverses mast cell infiltration, neural networks, angiogenesis and epithelium denudation in IC bladders. Furthermore, MSCs participate in regulating the self‐renewal, proliferation and differentiation of epithelial tissue‐resident stem cells in the basal layer of the urothelium to maintain epithelial integrity when urothelial damage induces bladder regeneration via regulation of sonic hedgehog (SHH) and WNT signalling pathways.^60^ Various growth factors, including the epidermal growth factor (EGF), insulin‐like growth factor (IGF) and FGF, can improve the differentiation of MSCs in a lipopolysaccharide (LPS)‐induced IC model by activating the SHH‐WNT signalling pathway; moreover, transplanted MSCs are differentiated into multiple cell types and gradually integrate into a perivascular‐like structure for up to 30 days.^61^ Furthermore, embryonic stem cell‐derived MSCs were differentiated into urothelium or pericytes of vascular endothelial tissue after activating WNT signalling pathways. The engrafted MSCs survived up to 6 months in rat bladders after injection in vivo and showed no signs of tumorigenesis.^62^

After engraftment into stromal and epithelial tissues, MSCs alleviate mast cell infiltration, inflammatory responses, angiogenesis, and denudation of the epithelium and subsequently decrease the voiding interval in IC models by activating the wingless/integrated (WNT) signalling.^63^ Intriguingly, gefitinib administration inhibited the therapeutic effects of MSCs by inhibiting WNT and epidermal growth factor receptor (EGFR) signalling activity, which indicates the importance of the key signalling pathway.^63^ Both MSC transplantation and phosphate buffered saline (PBS) injection promote vascular structure regeneration in IC models, while MSC transplantation alleviates submucosal oedema and haemorrhage. After localization to the bladder epithelium, MSCs were proven to decrease the concentrations of myeloperoxidase and the levels of inflammatory cytokines such as IL‐1β, IL‐6 and TNF‐α but increase the levels of fibroblast growth factor (FGF)‐2, vascular endothelial growth factor (VEGF), and the C‐C and C‐X‐C families of chemokines. Consequently, MSCs prolong the intercontraction interval and reduce nociceptive behaviours.^64^ Meanwhile, MSC transplantation promotes the regeneration of impaired bladder and improves the bladder voiding function in an IC rat model by downregulating the levels of interleukin (IL)‐1β, IL‐6 and TNF‐α and inhibiting the infiltration of mast cells and the apoptosis of urothelium.^65^ Xu et al.^66^ were the first to demonstrate that MSCs effectively mitigate inflammation of mast cells in the disease setting of IC/BPS. MSCs significantly improve the bladder micturition function after upregulation of antioxidants and antiapoptotic proteins, including haem oxygenase (HO)‐1, NAD(P)H quinine oxidoreductase (NQO)‐1 and B‐cell lymphoma 2 (Bcl‐2). Moreover, MSCs decrease the levels of the autophagy‐related protein LC3A and inflammatory factors (IL‐6, TNF‐α, nuclear factor kappaB [NF‐κB], caspase‐3 and Bcl‐2‐associated x [Bax]) in IC models.^67^ In addition, MSCs block ketamine‐induced injury in bladders by alleviating the mast cell infiltration, cellular apoptosis and fibrogenesis and subsequently alleviating defective bladder functions such as increased maximum contraction pressure, increased irregular voiding frequency and decreased intercontraction intervals and bladder capacity. Moreover, the levels of TGF‐β signalling‐related genes and phosphorylation of mothers against decapentaplegic homolog (SMAD)‐2 and SMAD‐3 proteins were downregulated by the MSC transplantation in IC rat models.^68^ Akira Furuta et al. reported that MSCs could decrease the levels of TNF‐α and transforming growth factor (TGF)‐β, number of mast cells, and accumulation of collagen fibres in the bladder of IC/BPS models. In addition, the MSC transplantation also significantly increased the bladder capacity, voiding threshold pressures and decreased nociceptive behaviour.^69^ MSCs remarkably increased the discharge volume, maximal micturition volume, contraction interval and bladder capacity while decreasing the residual urine volume, bladder pressure and bladder peak pressure in urinary bladder transplantation rat models by downregulating the TGF‐β/mitogen‐activated protein kinase (MAPK) signalling pathway and the levels of inflammatory cytokines and increasing the growth rate of local cells. Moreover, TGF‐β/MAPK signalling is termed the key pathway in MSC‐based therapy on urinary control and IC after urinary bladder transplantation.^70^ We have found that the anti‐inflammatory and immunoregulatory capacities of MSCs are more important and widely studied after we take a panoramic view of the situation from the current studies.

### 4.2 MSCs effectively alleviated tissue injury of partial BOO

Partial BOO is a common pathological condition induced by several lower urinary tract diseases, such as benign prostatic hyperplasia and urethral stricture. Moreover, partial BOO easily results in changes in structure and function in the bladder, which subsequently induce voiding dysfunction, detrusor overactivity, vesicoureteral reflux, urinary tract infection and overactive bladder.^71^ As we mentioned, BOO is a concomitant syndrome in female IC/BPS patients. MSC transplantation also significantly increased bladder function in BOO patients according to current studies. Partial BOO is known to downregulate the expression of IL‐10 while upregulating the expression levels of hypoxic, inflammatory and profibrotic markers, which subsequently induces the generation of smooth muscle hypertrophy and fibrosis in the bladder. Intriguingly, MSC transplantation significantly reduces the urine storage pressure, hypertrophy and fibrosis in the bladder via their immunomodulatory effects after 4 weeks. The related mechanism is that engrafted MSCs most predominantly reduce the TNF‐α levels and increase the IL‐10 and VEGF levels after 2 weeks.^72^ In addition, Chen et al. showed that the MSC transplantation significantly increased the average intravesical pressure and detrusor contraction power in ischaemia‐induced BOO models after MSCs were engrafted and regenerated in bladder tissue. They also show that MSCs increased the percentage of smooth muscle content and number of nerve fibres per high‐power field in rats with ischaemic bladder.^73^

### 4.3 Effects of MSCs from different origins

Multiple studies have demonstrated that MSCs from different origins have different therapeutic effects in other diseases, and the superior origin of MSCs may be attributed to the detailed disease type, since the surrounding microenvironment may influence the phenotype of MSCs.^74^, ^75^ In addition, MSCs isolated from different tissue regions differ in their properties.^76^ Likewise, Chung et al. proposed that urine‐derived stem cells had the best anti‐inflammatory effects compared to MSCs from other tissues, such as adipose tissue, bone marrow and amniotic fluid. Moreover, the best route was injection via bladder submucosa when compared with injection via tail vein or transurethral instillation, since MSC transplantation via bladder submucosa more significantly promotes the regeneration of damaged bladder tissue and recovery of bladder function by inhibiting inflammation accumulation.^11^ Another study by Choo et al. showed that the efficacy of human ESC‐derived MSCs and the survival time were the highest in treating IC in animal models in comparison with MSCs from umbilical cord blood, bone marrow and adipose tissue because ESC‐derived MSCs have higher engraftment.^62^ However, few studies compared different MSC origins, and they are not sufficiently powerful to finally determine the best tissue source.

### 4.4 Other treatments synergistically improve the effects of MSC transplantation in IC/BPS

Although MSCs are effective in alleviating bladder injury in IC/BPS animal models or patients, it is still necessary to improve the effects of MSCs in combination with other treatments (Table 2). Jung et al. demonstrated that N‐acetylcysteine (NAC) synergistically improves the therapeutic effects of MSCs in LPS‐induced IC rat models by protecting against inflammation and apoptosis in MSCs. The combination of MSCs and NAC was superior to monotherapy, since the combined therapy more significantly improved the contraction intervals and urination volume while reducing the residual volume.^77^ Chen et al. proved that melatonin improved the therapeutic effects of MSCs via its anti‐inflammatory and antioxidative stress effects in treating cyclophosphamide‐induced acute IC. The combined regimen of melatonin and MSCs more significantly decreased the 24‐hour urine volume, urine albumin level and severity of haematuria in acute IC rats.^78^ The combination of pentosan polysulfate treatment and MSC transplantation prolonged the intercontraction intervals but decreased the pain scores of IC models after they decreased mast cell infiltration, promoted urothelium regeneration and inhibited tissue fibrosis in bladder tissue. Moreover, pentosan polysulfate and MSCs synergistically decreased the levels of TNF‐α, IFN‐γ, monocyte chemotactic protein (MCP), IL‐6, Toll‐like receptor (TLR)‐2 and TLR‐11 compared with pentosan polysulfate treatment or MSC transplantation alone.^79^ Therefore, MSC transplantation should be considered an additional therapy in combination with current standard treatments. However, the detailed combination method requires further investigation in larger randomized trials to verify the efficacy and mechanisms.

**TABLE 2:** Combination of MSC transplantation and other treatments alleviates tissue injury in IC/BPS

Species	Tissue origin	Dose (MSC)	Combination	Control	Route	Model	Injury factor	Effect	Mechanism	Ref.
Human	Embryonic stem cell	2.5 or 5 × 10^4^	N‐Acetylcysteine	PBS	Outer layer of the bladder	Rat	PS/LPS	Increase contraction intervals; increase urination volume; reduce the residual volume	Relieve tissue inflammation	^77^
Rat	Adipose	1.2 × 10^6^	Melatonin	Saline	Intravenous	Rat	CYP	Decrease urine volume, urine albumin level, and severity of haematuria	Decrease the levels of inflammatory factors, glycosaminoglycan, oxidized protein, and reactive oxygen species; increase cellular expressions of antioxidants	^78^
Human	Adipose	1 × 10^6^	Pentosan polysulfate sodium	PBS	Bladder submucosa	Rat	Uroplakin3A	Prolong the intercontraction intervals; decrease the pain scores	Decrease the levels of TNF‐α, IFN‐γ, MCP, IL−6, TLR−2, and TLR−11	^79^

## 5 MSC‐DERIVED EXTRACELLULAR VESICLES AND IC/BPS

Mesenchymal stem cells can secrete a wide range of soluble factors into the culture medium, which are secretomes with lower immunogenic and tumorigenic potential.^80^ The detailed components of MSC‐derived extracellular vesicles vary depending on the original tissue source and in vitro external stimuli.^80^ These extracellular vesicles are classified into three types according to different diameters. Exosomes are 40–150 nm and formed by the endocytic pathway; microparticles are 50–1000 nm and are produced by the outward blebbing of the plasma membrane and subsequent release after the proteolytic cleavage of the cytoskeleton. Apoptotic bodies are 500–2000 nm and formed as by‐products of programmed cell death.^81^ Among them, MSC‐derived exosomes are the most widely used components for tissue repair and regenerative medicine according to current studies. Moreover, MSC‐derived exosomes horizontally transfer a large amount of validated therapeutic agents, such as nucleic acids, proteins, microRNA (miRNA) and lipids, into target cells to regulate cellular functions.^82^ Because of the abundant therapeutic agents, MSC‐derived exosomes can promote tissue regeneration by promoting the proliferation but inhibiting the apoptosis of endogenous cells.^83^, ^84^ In addition, MSC‐derived exosomes can maintain inner immune homeostasis by regulating the fate decision of related immune cells and attenuating excessive inflammation.^83^, ^85^ MSC‐derived exosomes also induce angiogenesis in injured tissue via stimulation of different signalling pathways.^86^, ^87^, ^88^

Mesenchymal stem cell‐derived extracellular vesicles exert dual effects, including regeneration and silencing inflammation on the urinary bladder wall, to prevent disease progression and recurrence. It is rich in growth factors and provides trophic stimulation in the bladder wall. Meanwhile, in vitro MSCs can secrete many cytokines with antiapoptotic, anti‐inflammatory, angiogenic and immunomodulative properties into the conditioned medium, which effectively promotes the regeneration of the urinary bladder for intravesical therapy in IC patients.^89^ Several studies clarified the therapeutic effects of MSC‐derived extracellular vesicles in coculture systems and in vivo studies. In a coculture system that consisted by human uroepithelial cells in the bottom compartment and MSCs in the upper compartment of a Transwell plate, MSCs significantly promoted the proliferation but inhibited the apoptosis of human uroepithelial cells treated with TNF‐α via activation of the serine/threonine kinase (AKT)/mechanistic target of rapamycin kinase (mTOR) signalling pathway.^65^ Hypoxia plays a key role in the progression of partial bladder outlet obstruction, since hypoxia significantly upregulates the levels of hypoxia‐inducible factor (HIF)‐3α, IL‐1β, IL‐6, TGF‐β1, TNF‐α, VEGF, alpha‐smooth muscle actin (α‐SMA), and total collagen expression and downregulates the expression level of IL‐10 in bladder smooth muscle cells (bSMCs). The direct coculture of MSCs and bSMCs takes effects in a cell‐to‐cell dependent manner, while the indirect coculture of MSCs and bSMCs takes effects in a cell‐to‐cell independent manner. Both direct and indirect MSC cocultures alleviate all inflammatory and profibrotic pathways in hypoxic bSMCs in vitro, which indicates that MSCs provide anti‐inflammatory and antifibrotic effects in a cell‐to‐cell independent manner.^90^ The family of basic fibroblast growth factors (bFGFs) identified in MSC‐derived exosomes are potent mitogens and enhance the proliferation and induction of urothelial cell proliferation in RAG1‐deficient mice.^91^ Freire et al^92^. demonstrated 31 differentially expressed miRNAs, and there was a direct correlation between miR‐449b, miR‐500, miR‐328 and miR‐320 and a downregulation of NK1R levels in IC/BPS patients. Lv et al.^93^ demonstrated that miR‐214 deficiency induced fibrogenesis in the bladder wall and resulted in IC in postmenopausal women via upregulation of mitochondrial fusion protein 2 (Mfn2) and promotion of epithelial‐mesenchymal transition. MSCs release miR‐214‐containing exosomes to suppress oxidative stress injury in cardiac stem cells through CaMKII silencing.^94^ Thus, miRNAs from MSC‐derived exosomes may be a new field in treating IC/BPS.

Based on available research findings, the profile of MSC‐derived extracellular vesicles appears to be an attractive tool to regulate the immune cell function in IC patients. The transplantation of MSC‐derived extracellular vesicles will effectively promote the repair of tissue injury in IC/BPS, and they take advantage of being cell‐free, having good biocompatibility, low antigenicity, and no cytotoxicity or oncogenicity. Considering the good efficacy of MSC‐derived exosomes and smaller diameters compared to the other two types of MSC‐derived extracellular vesicles, MSC‐derived exosomes require further study in larger randomized trials, with subgroup analysis to determine their management pathway of IC/BPS.

## 6 CONCLUSIONS

Bladder pain syndrome is a complex and chronic condition in which the exact aetiology has not been fully explored. Although there are various treatment tools for IC/BPS patients, most of them are not acknowledged as infallible options. We highlighted that MSCs and exosomes served as alternative and effective treatments because of their specific features, including powerful anti‐inflammatory and immunoregulatory capacities compared to other treatments. In addition, MSCs can engraft into bladder tissue and differentiate into key bladder cells, including urothelium and bSMCs, to promote bladder regeneration in IC/BPS. The transplantation of MSCs via different routes can effectively reverse bladder injury regardless of the detailed route according to current studies. Activation of several key signalling pathways such as AKT, mTOR, ERK, SHH and WNT is indispensable in MSC‐mediated bladder regeneration. However, there are limits in MSC‐based therapy in IC/BPS patients according to current studies. Most IC/BPS animal models were induced by a short acute inflammatory response but not chronic inflammation, which could not represent the real characteristics of IC/BPS patients. This result indicates that the potential mechanisms of IC/BPS and efficacy of MSC‐based therapy should be determined in an orthodox animal model. Second, some studies report that the effects of MSCs may be attributed to the origin. It is necessary to identify the ideal source of MSCs to treat IC/BPS. Third, the best dosage and injection route are still undetermined according to current MSC‐based experimental studies. It is meaningful to solve all these problems to seek optimized MSC‐based therapy in treating IC/BPS. Finally, MSC‐derived exosomes contribute to the treatment of IC/BPS without tumorigenicity, while whether MSC exosomes have comparable therapeutic efficacy to MSCs remains undetermined. Although MSC‐based therapy is still at a very early stage for urological practice and these issues should be resolved before clinical studies, we anticipate that further clarification of the related pathophysiological mechanisms of IC/BPS will contribute to guide the direction of MSC‐based treatments in the future.

## CONFLICTS OF INTEREST

The authors declare that they have no conflict of interest.

## AUTHOR CONTRIBUTIONS

**Chao Wen:** Investigation (lead); Resources (lead). **Liping Xie:** Validation (lead). **Chenxia Hu:** Conceptualization (lead); Supervision (lead).
